# First Molecular Survey and Phylogenetic Identification of a Zoonotic Pathogen, *Anaplasma platys*, in Hard Ticks Infesting Dogs in Southern Taiwan

**DOI:** 10.3390/vetsci13080759

**Published:** 2026-07-30

**Authors:** Li-Lian Chao, Chien-Ming Shih

**Affiliations:** 1 M.Sc. Program in Tropical Medicine, College of Medicine, Kaohsiung Medical University, Kaohsiung 807378, Taiwan; d91632003@gmail.com; 2 Graduate Institute of Medicine, College of Medicine, Kaohsiung Medical University, Kaohsiung 807378, Taiwan

**Keywords:** zoonotic pathogen, *Anaplasma platys*, tick, dog, Taiwan

## Abstract

*Anaplasma platys* was recognized as a zoonotic pathogen for canine and human infections. However, there is no confirmed evidence demonstrating the existence of *A. platys* within hard ticks (*Rhipicephalus sanguineus* sensu lato) infesting dogs in Southern Taiwan. We conducted a field survey for the detection of *Anaplasma* infection by screening the *R. sanguineus* s. l. ticks collected from dogs. Molecular detection was performed via a nested-PCR assay targeting the 16S rRNA gene of *Anaplasma*. Phylogenetic analyses were performed via neighbor-joining against maximum parsimony methods. The genetic relatedness was analyzed via pairwise distance calculation, using the Kimura 2-parameter model. The *A. platys* infection was detected in various life stages of *R. sanguineus* s. l. ticks, with an infection rate of 0.8%, 1.0% and 1.4% in nymphs, males and females, respectively. Monthly prevalence was observed from July to October. The genetic identity of detected *Anaplasma* strains from Taiwan was phylogenetically affiliated to the *A. platys* species with a 100% sequence homology verified from different countries. This study reveals the first molecular evidence of a zoonotic pathogen, *A. platys*, detected in *R. sanguineus* s. l. ticks from Southern Taiwan. Further investigation focused on the vectorial capacity of *R. sanguineus* s. l. ticks for transmitting *A. platys* will be beneficial to reveal the public health importance regarding the potential risk for canine and human infection in Southern Taiwan.

## Simple Summary

*Anaplasma platys* is a zoonotic pathogen affecting canines and humans. Although the hard ticks of *Rhipicephalus sanguineus* sensu lato largely infest dogs in Southern Taiwan, there is no evidence confirming *A. platys* infection in these ticks. We conducted a field survey for screening the *Anaplasma* infection in *R. sanguineus* s. l. ticks by nested-PCR assay. Results reveal infection rates of 0.8%, 1.0% and 1.4% in nymphs, males and females, respectively. Monthly prevalence was observed from July to October. Phylogenetic analysis demonstrating the detected *Anaplasma* spp. from Taiwan was genetically affiliated to the *A. platys* species, with a 100% sequence homology verified from different countries. Our results provide the first molecular evidence identifying the *A. platys* detected in *R. sanguineus* s. l. ticks in Southern Taiwan and highlight the potential threat for canine and human infections in Southern Taiwan.

## 1. Introduction

The hard tick *Rhipicephalus sanguineus* has been observed as the most common tick species infesting dogs [1]. This tick species has three life-cycle stages (i.e., larval, nymph and adult), with each stage engorging blood from the infested host. The geographical distribution of *R. sanguineus* has been recorded throughout the tropical and subtropical countries [2,3]. Current studies based on the mitochondrial 16S ribosomal DNA have revealed the existence of at least two separate groups (tropical vs. temperate lineage) of *R. sanguineus* ticks [4,5,6] and are currently treated as *R. linnaei* and *R. sanguineus* sensu stricto, respectively. In our previous study, the *R. sanguineus* ticks collected from Southern Taiwan were genetically affiliated with the tropical lineage of *R. sanguineus* sensu lato [7], and this species is currently treated as *R. linnaei* [8]. In addition, the *R. sanguineus* complex was recognized as the main vector tick for various zoonotic pathogens, including *Babesia gibsoni*, *B. vogeli*, *Hepatozoon canis*, *Rickettsia conorii*, *R. massiliae*, *R. rickettsia*, *Ehrlichia canis* and *Anaplasma platys* that are transmitted among canine hosts [9,10,11,12,13,14]. In Taiwan, this tick species was observed as the dominant tick species infesting dogs in northern Taiwan [15]. Although *R. sanguineus* has been detected with various zoonotic pathogens, including *Rickettsia*, *Babesia* and *Ehrlichia* in Taiwan [7,16,17,18,19], there is no confirmed evidence demonstrating the existence of *A. platys* within *R. sanguineus* ticks collected from Southern Taiwan.

The genus *Anaplasma* is an intracellular bacterium that includes *A. phagocytophilum*, a species infecting numerous mammals and humans; the ruminant pathogens including *A. centrale*, *A. ovis*, and *A. bovis*; and the canine pathogen, *A. platys* [20,21]. In general, the hard ticks of Ixodidae served as common vectors and reservoir hosts for the agent of *Anaplasma* spp. [19,21,22,23,24,25,26]. In previous reports, *A. platys* has been recognized as the etiological agent for zoonotic infection of dogs in various countries [13,27,28,29,30], and human infections with *A. platys* have also been described [31,32]. Although a serosurvey of *Anaplasma* and *Ehrlichia* infections in dogs has been investigated in Central Taiwan [33], there is no report confirming the vector ticks responsible for the transmission of *A. platys*. Thus, a molecular survey for *A. platys* in *R. sanguineus* ticks is essential for evaluating the possible risk regarding *A. platys* infections for canine and human populations in Southern Taiwan.

Currently, there is no report providing confirmed evidence of *A. platys* in hard ticks of *R. sanguineus* s. l. in Southern Taiwan. Accordingly, the aims of this study were designed to screen the existence of *Anaplasma* spp. in these hard ticks parasitizing dogs of Southern Taiwan and to determine the genospecies of *Anaplasma* spp. within these ticks. In addition, phylogenetic analysis was also performed to display the genetic relationship of *Anaplasma* spp. detected in these ticks in Southern Taiwan.

## 2. Materials and Methods

### 2.1. Tick Collection and Species Identification

**Figure 1:**
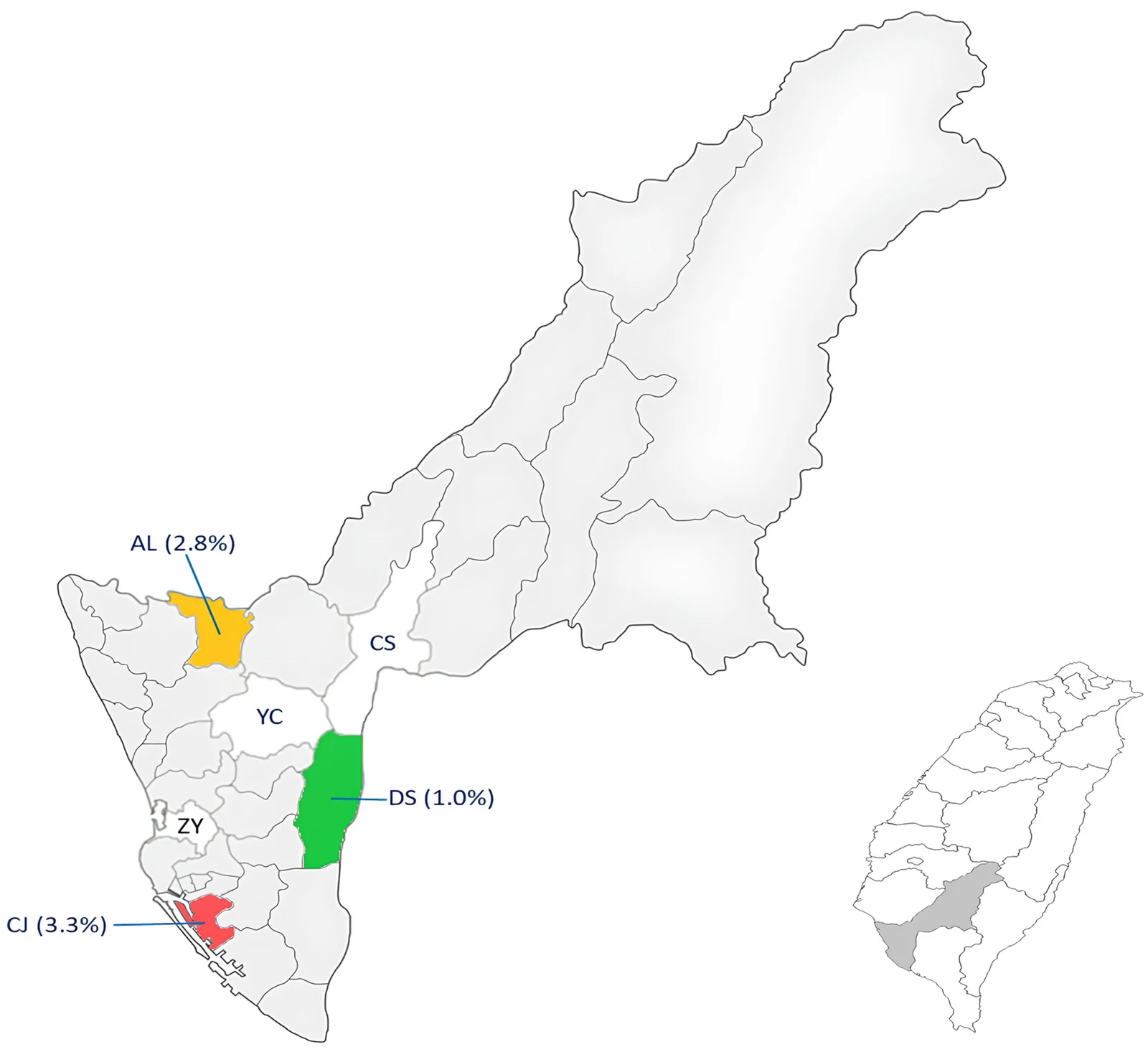
Map of Taiwan showing the tick collection from six districts of Kaohsiung City and the percentage of *Anaplasma platys* infection detected in ticks from DS (Da-Shu), AL (A-Lian), CJ (Cian-Jhen), Zuo-Ying (ZY), Yan-Chao (YC) and Ci-Shan (CS) districts of Kaohsiung City, Taiwan.

Tick specimens infesting stray dogs that appeared to be healthy were collected monthly for one year from six districts of Kaohsiung City, including Da-Shu (DS), Yan-Chao (YC), Zuo-Ying (ZY), Cian-Jhen (CJ), Ci-Shan (CS) and A-Lian (AL), located in Southern Taiwan (Figure 1). To avoid interference from blood, only flat (unengorged) ticks were selected for molecular screening of *Anaplasma* spp. infection. A total of 1512 ticks (504 females, 503 males and 505 nymphs) were collected, and the collected ticks were cleaned using sonication in distilled water and subsequently stored in 75% ethanol in glass vials. The tick specimens were identified to the species level according to the pictorial keys of *Rhipicephalus*, as described previously [34,35]. In general, a Nikon stereo-microscope (SMZ 1500, Tokyo, Japan) was used to observe the external structures of these ticks and to photograph the features for species identification, as described previously [18].

### 2.2. DNA Extraction from Tick Specimens

Total genomic DNA was extracted from each collected tick. In general, tick specimens were cleaned by 3–5 min sonication with 75% ethanol solution and then washed twice in sterile distilled water. Thereafter, each tick specimen was homogenized with 180 μL lysing buffer solution by a TissueLyser II apparatus (catalogue no. 85300, Qiagen, Hilden, Germany). The resulting homogenate was centrifuged at room temperature, and the supernatant fluid was further processed by a commercial kit (catalogue no. 69506, DNeasy Blood & Tissue Kit, Qiagen, Taipei, Taiwan). The DNA-containing filtrated fluid was collected and quantitated with a spectrophotometer (Epoch, Biotek, Winooski, VA, USA). All the quantitated DNA specimens were preserved in a deep freezer (−80 °C) until further analysis, as described previously [17].

### 2.3. Molecular Detection of Anaplasma by Nested Polymerase Chain Reaction (nPCR)

The nPCR assays were conducted to screen DNA samples extracted from individual tick specimens by targeting the 16S rRNA gene of *Anaplasma*. The PCR amplification used two primer sets, including the primer set of ECC/ECB for the initial amplification and the primer set of EPLAT5/EPLAT3 targeting the species-specific DNA fragment of *A. platys*, which was used for the secondary amplification, producing a DNA product approximately 359 bp in length, as described previously [36,37]. The Taq polymerase and PCR reagents were commercially purchased and used as instructed (Takara Shuzo Co., Ltd., Tokyo, Japan). In general, each PCR reaction contains a 25 μL reaction mixture that includes 3 μL DNA template, 2 μL dNTP mixture (10 mM each), 2.5 μL 10× PCR buffer (Mg^2+^), 1.5 μL forward and reverse primers, 1 unit of Taq DNA polymerase, and the remaining volume was replenished with ddH_2_O, as described previously [38]. In parallel with each reaction, the reaction mixture was supplemented with adequate amounts of sterile distilled water and used as a negative control.

The entire PCR process was performed by a Veriti thermocycler (Applied Biosystems, Taipei, Taiwan) under the following conditions: initial denaturation at 94 °C for 3 min, followed by 35 cycles with the specific conditions of denaturation at 94 °C for 1 min, annealing at 55 °C for 2 min and extension at 72 °C for 2 min. For secondary PCR assay, the same initial denaturation was performed and followed by 35 cycles with the same denaturation and annealing conditions, except the extension condition (72 °C for 90 s), followed by a final extension step (72 °C for 2 min). All amplified PCR products were examined by electrophoresing on 1.5% agarose gels after staining with ethidium bromide and observed under ultraviolet light. A standard DNA marker (100 bp ladder, GeneRuler, Thermo Scientific, Taipei, Taiwan) was used to measure the molecular size of the amplified PCR products.

### 2.4. Gene Sequencing and Phylogenetic Analysis

For gene sequencing, each selected sample (approximately 10 μL) appearing with clear band on agarose gel was prepared and submitted for sequencing (Mission Biotech Co., Ltd., Taipei, Taiwan). Briefly, the same primer sets and conditions of nested-PCR were used for the sequencing reaction with an ABI Prism 377-96 DNA Sequencer (Applied Biosystems, Foster City, CA, USA) using the Big Dye Terminator Cycle Sequencing Kit, as described previously [37]. The resulting sequences were edited and aligned with software by BioEdit (V5.3) and CLUSTAL W (version X), respectively [39].

For phylogenetic analysis, the aligned 16S rRNA gene sequences from 6 *Anaplasma* spp. of Taiwan were compared by analyzing with other 17 strains of *Anaplasma* spp., *Rickettsia* spp. and *E. coli* verified from various geographical and biological origins documented in GenBank (Table 1). The phylogenetic analyses were performed with neighbor-joining (NJ) and maximum parsimony (MP) methods by estimating the entire alignment using MEGA X software package [40]. The genetic distance values were also analyzed by the Kimura two-parameter model to demonstrate the inter- and intra-species variations [41]. The phylogenetic trees were constructed and the reliability of the construction was evaluated with 1000 bootstrap replications, as described previously [42].

### 2.5. Nucleotide Sequences Registration for Anaplasma Detected in Ticks of Southern Taiwan

The nucleotide sequences of 16S rRNA gene of 6 *Anaplasma* spp. detected in hard ticks of *R. sanguineus* s. l. from Southern Taiwan were submitted to GenBank and assigned the following accession numbers: PX285921, PX307904, PX307910, PX307909, PX307921 and PX285925, as indicated in Table 1. In addition, the 16S rRNA gene sequences from 17 strains (fourteen *Anaplasma* spp. and three outgroup spp.) were also included for comparison (Table 1).

### 2.6. Statistical Analysis

The detection rate of *A. platys* infection in different life stages of ticks and various districts of Kaohsiung City was analyzed by Chi-square test (SPSS 20 software).

## 3. Results

### 3.1. Molecular Detection of Anaplasma platys in Dog Ticks from Southern Taiwan

The nested PCR assay targeting the 16S rRNA gene was used to detect the existence of *A. platys* in *R. sanguineus* ticks. *A. platys* was detected in *R. sanguineus* s. l. ticks collected from three districts of Kaohsiung City (Table 2). The highest geographical prevalence of *A. platys* was detected in the Cian-Jhen district (3.3%), followed by A-Lian (2.8%) and Da-Shu (1.0%) (Table 2). Based on the life-cycle stage of ticks, the *A. platys* infection was detected in females, males and nymphs with infection rates of 1.4%, 1.0% and 0.8%, respectively (Table 2). The detection rate of *A. platys* infection was significantly higher in the Cian-Jhen district (Chi-square test, *p* < 0.05) (Table 2). However, the detection rate of *A. platys* infection in females is insignificantly higher than that in males or nymphs (Chi-square test, *p* > 0.05) (Table 2).

### 3.2. Monthly Prevalence of A. platys Infection in Dog Ticks from Southern Taiwan

**Figure 2:**
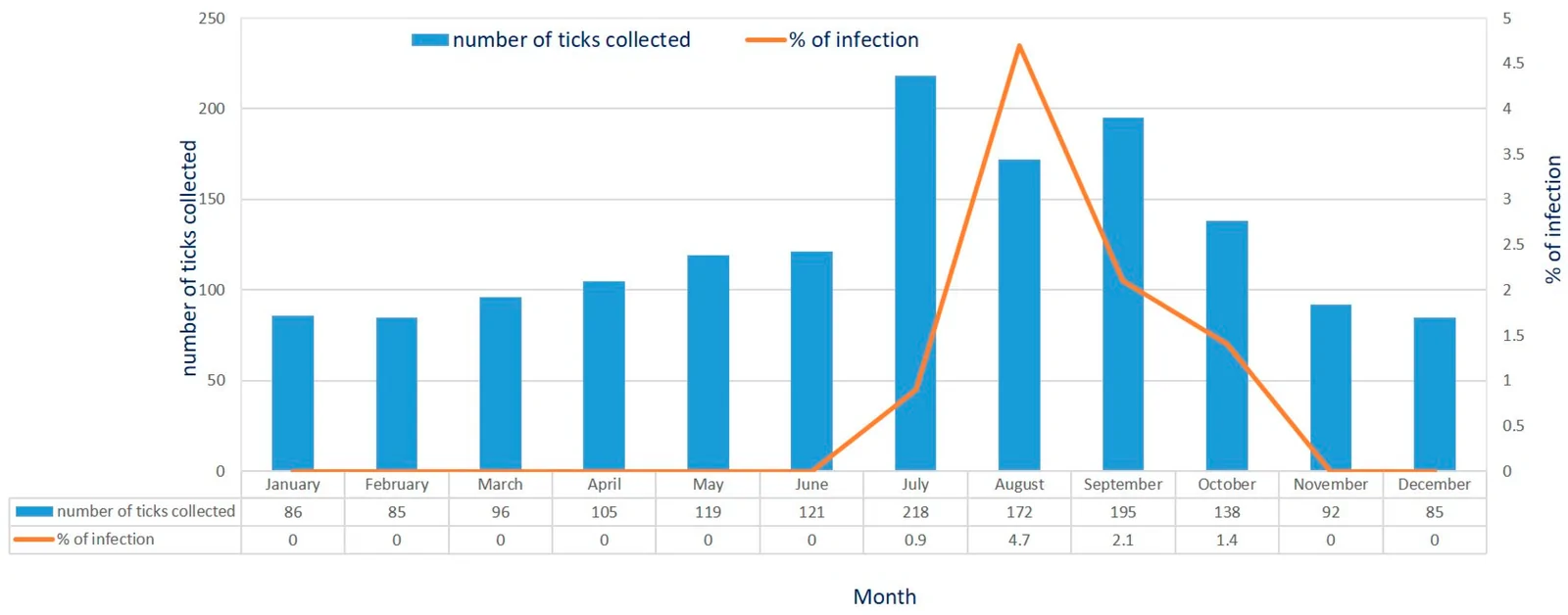
Monthly collection of *Rhipicephalus sanguineus* s. l. ticks from dogs and percentage of infection of *Anaplasma platys* in these ticks from Southern Taiwan.

The detection rate of *A. platys* infection was measured from July to October and dramatically increased during the Summer (July–August) and Autumn (September–October) seasons. The highest detection rate was in August, with a detection rate of 4.7%, followed by detection rates of 2.1%, 1.4% and 0.9% in September, October and July, respectively (Figure 2).

### 3.3. Genetic Identity of Anaplasma spp. Detected in Dog Ticks of Southern Taiwan

To identify the genetic identity of *Anaplasma* spp. detected in *R. sanguineus* s. l. ticks of Southern Taiwan, 16S rRNA gene sequences of six *Anaplasma* strains from Taiwan were compared with fourteen other *Anaplasma* spp. and three outgroup strains verified from various geographical and biological origins. Results indicated that the genetic relatedness of all *Anaplasma* spp. detected in *R. sanguineus* s. l. ticks of Taiwan was affiliated to the genospecies of *A. platys* detected in China (GenBank no: OK639189) and Thailand (GenBank no: PV875279) with a 100% sequence similarity (Table 3). Furthermore, inter- and intra-species analysis based on the genetic distance (GD) values of the 16S rRNA gene revealed no distance (GD = 0.00) within the *Anaplasma* strains of Taiwan, as compared with a high level of genetic divergence with the other species of *Anaplasma* (GD > 0.02) and outgroup species (GD > 0.16) (Table 3).

### 3.4. Phylogenetic Affiliation of Anaplasma spp. Detected in Dog Ticks of Southern Taiwan

**Figure 3:**
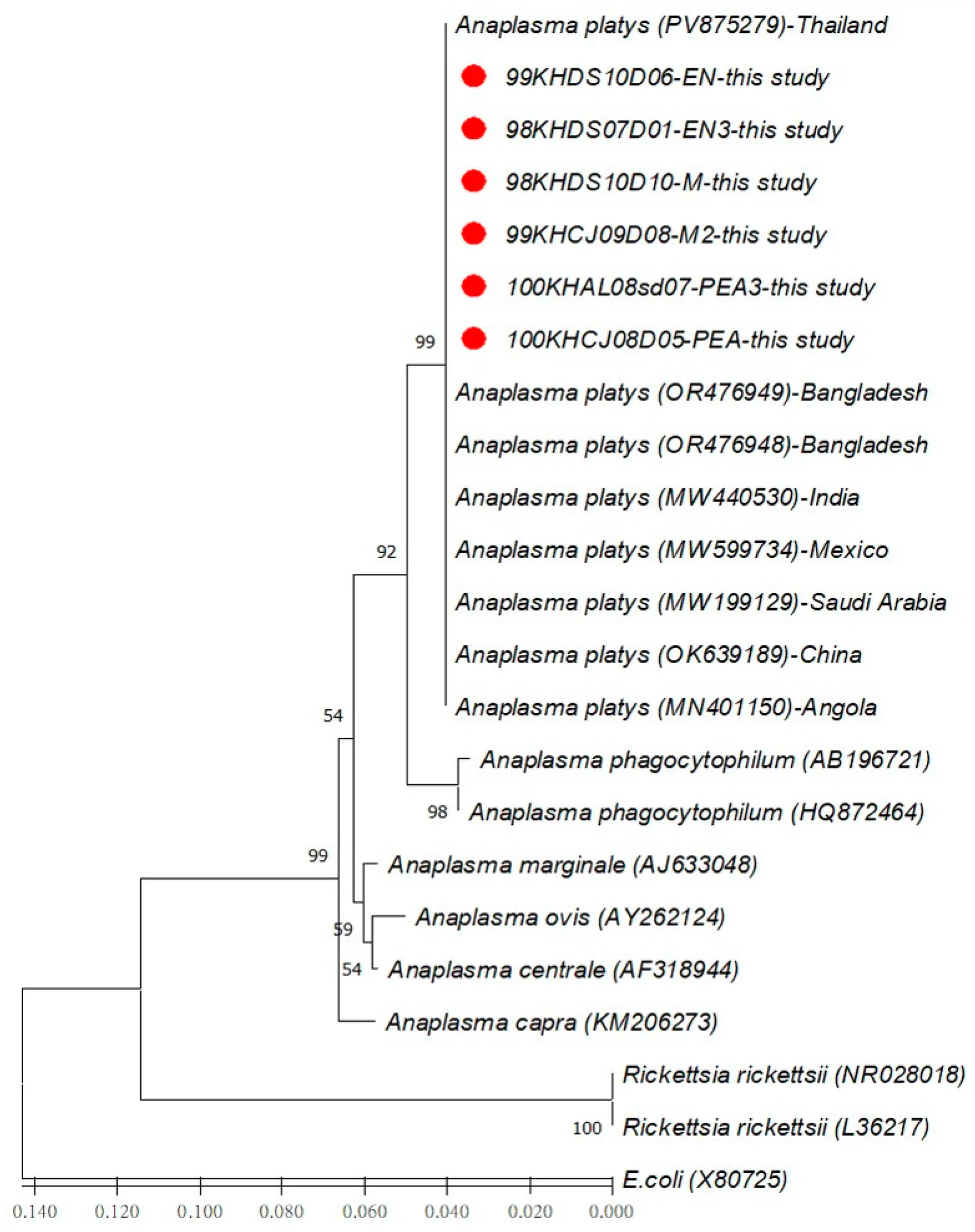
Phylogenetic tree constructed by neighbor-joining (NJ) method based on the 16S rRNA gene. The aligned sequences of six *Anaplasma* strains (indicated as ●) detected in *Rhipicephalus sanguineus* s. l. ticks from Kaohsiung City of Taiwan were analyzed with fourteen other *Anaplasma* strains and three outgroup strains verified from various geographical and biological origins. The tree was analyzed using 1000 bootstrap replicates, and the numbers at the nodes display the percentages of reliability. Branch length is drawn proportional to the estimated sequence divergence.

**Figure 4:**
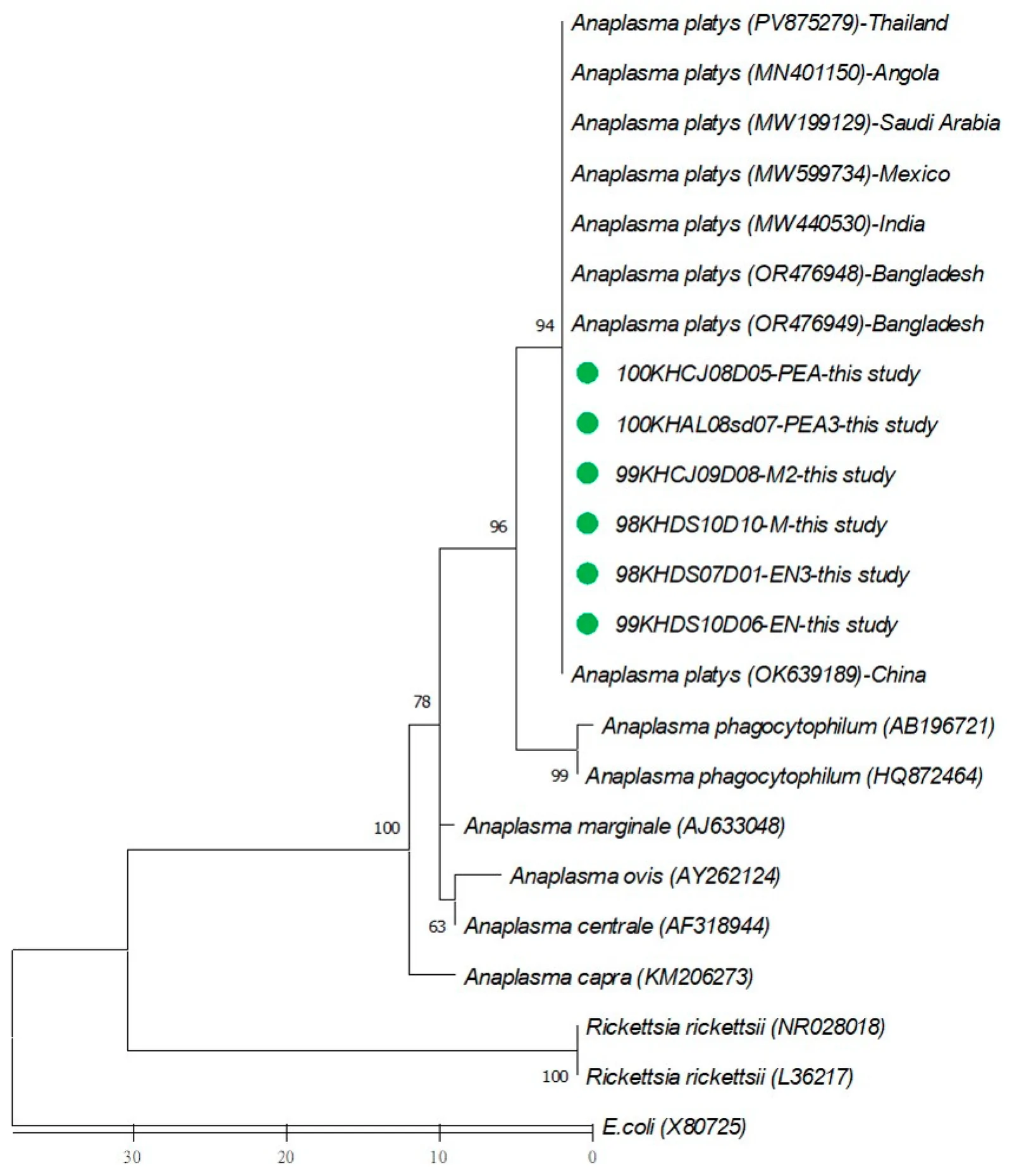
Phylogenetic tree constructed by maximum parsimony (MP) method based on the 16S rRNA gene. The aligned sequences of six *Anaplasma* strains (indicated as ●) detected in *Rhipicephalus sanguineus* s. l. ticks from Kaohsiung City of Taiwan were analyzed with fourteen other *Anaplasma* strains and three outgroup strains verified from various geographical and biological origins. The tree was analyzed using 1000 bootstrap replicates, and the numbers at the nodes display the percentages of reliability. Branch length is drawn proportional to the estimated sequence divergence.

The phylogenetic relationships among 20 species of *Anaplasma* and three outgroup species were analyzed by constructing the phylogenic trees using neighbor-joining (NJ) and maximum parsimony (MP) methods. Results indicated five major clades of *Anaplasma* spp. displayed in both NJ (Figure 3) and MP (Figure 4) trees, which can be easily distinguished between different *Anaplasma* species and outgroup species. Briefly, a monophyletic clade constituted by all *Anaplasma* species detected in this study was genetically affiliated to the genospecies of *A. platys* identified from various countries. These results demonstrate a high genetic homology (100% sequence similarity) within the *A. platys* strains detected in *R. sanguineus* ticks of Southern Taiwan, but a higher genetic divergence (high sequence variation) distinguished them from other groups of *Anaplasma* species identified from different geographical and biological origins (Figure 3 and Figure 4).

## 4. Discussion

Our results demonstrate the primary prevalence and genetic characterization of *Anaplasma* species detected in hard ticks (*R. sanguineus* s. l.) from Southern Taiwan. The genetic analysis reveals that these *Anaplasma* species are phylogenetically affiliated to the genospecies of *A. platys,* which was first identified in hard ticks (*R. sanguineus* s. l.) of Southern Taiwan. In previous reports, *A. platys* was first verified as an etiological agent for the cyclic thrombocytopenia of canine and is described to infect the host’s platelets [43,44]. This canine infection was reported throughout the world and was characterized by mild clinical symptoms in infected dogs [27,28,45,46,47]. However, canine infection with severe clinical symptoms has mainly been reported in Europe [48,49,50]. Although the *R. sanguineus* s. l. tick is incriminated as the major tick species responsible for the zoonotic transmission of *A. platys* among canines, this zoonotic pathogen (*A. platys*) has also been identified in various hard ticks, such as *Ixodes persulcatus*, *Haemaphysalis longicornis* and *Dermacentor auratus* [45,51]. Nevertheless, our study provides the first molecular evidence identifying *A. platys* in hard ticks (*R. sanguineus* s. l.) in Southern Taiwan.

The monthly variation in *A. platys* infection in hard ticks (*R. sanguineus* s. l.) of Southern Taiwan remains uncertain. Our results reveal the prevalence of *A. platys* infection in hard ticks (*R. sanguineus* s. l.) detected from July to October, and the highest detection rate was observed in August (Figure 1). During the hot months, this observation is coincident with the monthly abundance of the tick population described in our previous investigation [15]. In addition, it has also been reported that the *R. sanguineus* s. l. ticks exposed to high temperatures attach more rapidly to animals and humans [52,53]. Indeed, the warmer climate during the long summer season can induce the attaching movement of *R. sanguineus* s. l. ticks for approaching animal hosts in the natural environment [54]. Thus, the abundance of tick populations in various months was highly correlated with the monthly prevalence of *A. platys* detected in hard ticks (*R. sanguineus* s. l.) of Southern Taiwan.

Molecular identification of the tick-borne pathogens by analyzing the sequences of the specific target gene makes it possible to measure the genetic variation and determine the genetic identity among *Anaplasma* strains. Indeed, previous investigations on *Anaplasma* spp. have revealed the validity of the 16S rRNA gene for identifying *A. platys* in ticks and dogs [13,27,28,49,55,56]. In addition, genetic comparison of the 16S rRNA gene sequences between the *A. platys* strains from dogs in different areas constitutes a separate cluster on the phylogenetic tree with a high homology [46,47,49]. Thus, molecular characterization according to the 16S rRNA gene of *Anaplasma* spp. provides a reliable tool for discriminating the genetic variation and determining the genetic identity of *Anaplasma* species within *R. sanguineus* ticks.

The phylogenetic relationship among *Anaplasma* spp. in hard ticks (*R. sanguineus* s. l.) can be distinguished by analyzing the sequence similarity based on the 16S rRNA genes of *Anaplasma*. In our investigation, genetic identification based on the phylogenetic analysis of 16S rRNA genes of *Anaplasma* indicated that all *Anaplasma* spp. detected in hard ticks (*R. sanguineus* s. l.) from Southern Taiwan display an obvious affiliation with the genospecies of *A. platys* (Figure 3 and Figure 4). These *A. platys* strains detected in hard ticks (*R. sanguineus* s. l.) of Southern Taiwan are highly associated with the *A. platys* strain verified from a dog from Thailand (GenBank no. PV875279) and an *R. sanguineus* tick from China (GenBank no. OK639189), evinced by 100% sequence identity (Table 3). This genetic discrimination is strongly supported by the phylogenic trees constructed by either NJ or MP analysis (Figure 3 and Figure 4). Thus, our results reveal the genetic identity of *Anaplasma* spp. detected in hard ticks (*R. sanguineus* s. l.) of Southern Taiwan as a monophyletic group affiliated with *A. platys*, a zoonotic pathogen for canine anaplasmosis.

The prevalence of *A. platys* infection in collected ticks may correlate with environmental and biological factors. In our results, the prevalence of *A. platys* infection was observed significantly higher in the Cian-Jhen (CJ) district (Table 2). Indeed, there are large municipal parks in this district, and these parks gather a high population of stray dogs that may contribute to the high abundance of ticks and high prevalence of *A. platys* infection in collected ticks. In addition, the prevalence of *A. platys* infection had been detected in 19.6% of *R. sanguineus* s. l. ticks and 15.4% of accompanying dog blood collected from Nantou County in central Taiwan [57]. These results are significantly higher than our detection in *R. sanguineus* s. l. ticks (0.8–1.4%) from Kaohsiung City of Southern Taiwan (Table 2). The variation in *A. platys* infection in *R. sanguineus* s. l. ticks between Nantou County and Kaohsiung City may be due to the geographical variation between the mountainous area of Nantou County and urbanized Kaohsiung City. Indeed, the mountainous area of Nantou County provides higher feeding activity for *R. sanguineus* s. l. ticks on canine hosts. Further investigation focused on the biting frequency of *R. sanguineus* s. l. ticks on canine hosts may help to illustrate this puzzle.

The actual mechanisms for the maintenance of *A. platys* in *R. sanguineus* ticks remain controversial. Results in this study reveal the detection of *A. platys* in nymph and male ticks of *R. sanguineus,* and the higher prevalence of *A. platys* in male ticks may be inherited from infected nymphs. Indeed, the previous study has reported that the transstadial transmission of *A. platys* infection was observed from infected nymphs to the adult stage of *R. sanguineus* ticks [11]. In addition, the transovarial transmission of *A. platys* infection through several generations was also observed in a laboratory colony of *R. sanguineus* ticks [58]. Furthermore, the co-feeding mechanism may also account for another mechanism responsible for the transmission of *A. platys* infection from an infected tick to a new tick by feeding closely on the same host [59]. Thus, the possibility of biological transmission of *A. platys* within *R. sanguineus* ticks needs to be further identified.

## 5. Conclusions

Our results reveal the initial detection of *A. platys* in hard ticks (*R. sanguineus* s. l.) infesting dogs in Southern Taiwan. According to the phylogenetic analyses, the genetic identity of *A. platys* was first identified in *R. sanguineus* s. l. ticks of Southern Taiwan. Human infections with *A. platys* had been reported in patients exposed to *R. sanguineus* s. l. ticks [31] and accompanied dog blood [32]. Further study focused on the prevalence of *A. platys* infection in accompanied canine hosts will benefit the evaluation of the transmission risk of *A. platys* infection among canines and illustrate the potential threat for human infection in Southern Taiwan.

## Figures and Tables

**Table 1:** Phylogenetic analysis of *Anaplasma* strains of Taiwan with other bacterial strains documented in GenBank based on 16S rRNA gene.

Genospecies/Strain	Origin of Bacterial Strain		GenBank Accession Number ^a^
	Biological	Geographic	
Taiwan strains			
98KHDS07D01-EN3	*Rhipicephalus sanguineus* sensu lato	Kaohsiung, Taiwan	**PX285921**
98KHDS10D10-M	*Rhipicephalus sanguineus* s. l.	Kaohsiung, Taiwan	**PX307904**
99KHDS10D06-EN	*Rhipicephalus sanguineus* s. l.	Kaohsiung, Taiwan	**PX307910**
99KHCJ09D08-M2	*Rhipicephalus sanguineus* s. l.	Kaohsiung, Taiwan	**PX307909**
100KHCJ08D05-PEA	*Rhipicephalus sanguineus* s. l.	Kaohsiung, Taiwan	**PX307921**
100KHAL08sd07-PEA3	*Rhipicephalus sanguineus* s. l.	Kaohsiung, Taiwan	**PX285925**
*Anaplasma platys*	Dog	Thailand	PV875279
*Anaplasma platys*	Dog	Angola	MN401150
*Anaplasma platys*	Dog	Saudi Arabia	MW199129
*Anaplasma platys*	Dog	Mexico	MW599734
*Anaplasma platys*	Dog	India	MW440530
*Anaplasma platys*	*Rhipicephalus sanguineus*	China	OK639189
*Anaplasma platys*	*Rhipicephalus sanguineus*	Bangladesh	OR476948
*Anaplasma platys*	Dog	Bangladesh	OR476949
*Anaplsma phagocytophilum*	Deer	Japan	AB196721
*Anaplsma phagocytophilum*	Goat	China	HQ872464
*Anaplasma marginale*	Cattle	China	AJ633048
*Anaplasma ovis*	Sheep	China	AY262124
*Anaplasma centrale*	Vaccine	The Netherlands	AF318944
*Anaplasma capra*	Homo sapiens	China	KM206273
*Rickettsia rickettsii*	Type strain	USA	NR028018
*Rickettsia rickettsii*	*Dermacentor andersoni*	USA	L36217
*Escherichia coli*	Neotype strain	France	X80725

^a^ The bold letters of GenBank accession numbers were submitted by this study.

**Table 2:** Molecular detection of *Anaplasma platys* in *Rhipicephalus sanguineus* s. l. ticks infesting dogs from various districts of Kaohsiung City in Southern Taiwan by nested-PCR assay.

District ^a^ of Tick Collection	*A. platys* Detected in Various Life Stages of Tick			Total
	Female	Male	Nymph	
	No. Infected /No. Examined (%)	No. Infected /No. Examined (%)	No. Infected /No. Examined (%)	No. Infected /No. Examined (%)
DS	0/134 (0)	2/153 (1.3)	4/291 (1.4)	6/578 (1.0) ^b^
YC	0/155 (0)	0/112 (0)	0/100 (0)	0/367 (0)
ZY	0/81 (0)	0/74 (0)	0/36 (0)	0/191 (0)
CJ	2/53 (1.9)	2/64 (3.1)	0/5 (0)	4/122 (3.3) ^b^
CS	0/11 (0)	0/14 (0)	0/11 (0)	0/36 (0)
AL	5/70 (7.1)	1/86 (1.2)	0/62 (0)	6/218 (2.8) ^b^
Total	7/504 (1.4) ^c^	5/503 (1.0) ^c^	4/505 (0.8) ^c^	16/1512 (1.1)

^a^ DS (Da-shu), YC (Yan-Chao), ZY (Zuo-Ying), CJ (Cian-Jhen), CS (Ci-Shan), AL (A-Lian). ^b^ Chi-square test, *p* = 3.86, *p* < 0.05. ^c^ Chi-square test, *p* = 0.83, *p* > 0.05.

**Table 3:** Intra- and Inter-species analysis of genetic distance values ^a^ based on the 16S rRNA gene sequences between *Anaplasma* strains detected in *Rhipicephalus sanguineus* s. l. ticks of Taiwan and other strains of *Anaplasma*, *Rickettsia* and *E. coli* documented in GenBank.

Bacterialial Strains ^b^ (GenBank No.)	1	2	3	4	5	6	7	8	9	10	11	12	13	14	15
1. *Anaplasma platys*-Thailand (PV875279)	—														
2. *Anaplasma platys*-China (OK639189)	0.00	—													
3. 100KHAL08sd07-PEA3-**Taiwan** (PX285925)	0.00	0.00	—												
4. 100KHCJ08D05-PEA-**Taiwan** (PX307921)	0.00	0.00	0.00	—											
5. 99KHCJ09D08-M2-**Taiwan** (PX307909)	0.00	0.00	0.00	0.00	—										
6. 99KHDS10D06-EN-**Taiwan** (PX307910)	0.00	0.00	0.00	0.00	0.00	—									
7. 98KHDS10D10-M-**Taiwan** (PX307904)	0.00	0.00	0.00	0.00	0.00	0.00	—								
8. 98KHDS07D01-EN3-**Taiwan** (PX285921)	0.00	0.00	0.00	0.00	0.00	0.00	0.00	—							
9. *Anaplasma phagocytophilum* (AB196721)	0.03	0.02	0.02	0.02	0.02	0.02	0.02	0.02	—						
10. *Anaplasma centrale* (AF318944)	0.03	0.03	0.03	0.03	0.03	0.03	0.03	0.03	0.03	—					
11. *Anaplasma marginale* (AJ633048)	0.03	0.03	0.03	0.03	0.03	0.03	0.03	0.03	0.03	0.01	—				
12. *Anaplasma ovis* (AY262124)	0.04	0.03	0.03	0.03	0.03	0.03	0.03	0.03	0.04	0.01	0.02	—			
13. *Anaplasma capra* (KM206273)	0.04	0.03	0.03	0.03	0.03	0.03	0.03	0.03	0.05	0.02	0.02	0.02	—		
14. *Rickettsia rickettsii* (NR028018)	0.19	0.19	0.19	0.19	0.19	0.19	0.19	0.19	0.18	0.16	0.16	0.17	0.17	—	
15. *Escherichia coli* (X80725)	0.25	0.24	0.24	0.24	0.24	0.24	0.24	0.24	0.25	0.24	0.24	0.24	0.23	0.29	—

^a^ The pairwise distance calculation was performed by the method of Kimura 2-parameter, as implemented in MEGA X [40]. ^b^ The *Anaplasma* strains 3–8 were detected in *Rhipicephalus sanguineus* ticks of Southern Taiwan.

## Data Availability

The raw data supporting the conclusions of this article will be made available by the authors on request.
